# Supplementary material for: The Effect of Illumination Patterns during Mung Bean Seed Germination on the Metabolite Composition of the Sprouts
Source: Plants (Basel). 2023 Nov 4;12(21):3772. doi: 10.3390/plants12213772 (PMC10649298; doi:10.3390/plants12213772)
Supplement: Supplementary file 1 [file plants-12-03772-s001.zip › plants-2651558-supplementary.pdf]

Supplementary Materials.

Table S1. The content of metabolite compounds in the dark-exposed sprouts (DS), ppm (mg/kg).

**Metabolite compound**	**Minimum**	**Maximum**	**Mean**	**Standard Error**
Oxalate	17	116	43	26
Lactic acid	49	347	114	71
3-Hydroxypropionic acid	4	11	8	2
Pyruvic acid	9	44	23	9
Succinic acid	33	156	100	29
Malic acid	176	1455	489	298
Fumaric acid	3	14	6	3
Tartaric acid	15	119	67	26
Citric acid	18	370	96	91
Glyceric acid	22	65	52	13
Methyl-glyceric acid	0	18	2	4
Threonic acid	39	247	157	61
Erythronic acid	7	22	17	5
Erythrono-1.4-lactone+threono-1.4-lacton	3	19	12	5
Ribonic acid	10	51	31	13
Gluconic acid	7	332	162	112
Gluconic-6-phosphate acid	0	12	3	4
2-Ketogluconic acid*			6	
Saccharic acid	9	48	25	13
Nicotinic acid	6	40	21	11
Shikimic acid	7	168	34	40
Quinic acid	0	504	40	134
Caffeic acid	37	333	88	75
4-Hydroxycinnamic acid	13	148	40	33
Benzoic acid	4	12	7	3
2.3-dihydrobenzoic acid*			2	
Dehydroabietic acid*			10	
Azelaic acid	0	16	4	7
Mesoxalic acid*			4	
Maleic acid*			14	
Phosphoric acid	824	1907	1295	318
Phosphate + methyl-phosphate	100	486	275	90
Valine	43	950	369	274
Leucine + Isoleucine	27	736	265	206
Methionine*			11	
Threonine	0	54	24	16
Phenylalanine	9	103	34	30
Tryptophan	0	22	4	7
Lysine + Histidine	52	288	100	63
Arginine + Citrulline	0	56	19	18
α-Alanine	27	108	65	22

Glycine	0	35	9	8
Proline	12	149	59	42
Serine	14	120	49	31
Tyrosine	0	70	8	18
Aspartic acid	134	608	325	138
Asparagine	33	740	291	232
Glutamic acid	72	443	227	111
Glutamine	0	99	18	26
Ornithine	0	207	37	64
Ornithine lactam	0	605	197	203
β-Alanine	0	10	3	4
Hydroxyproline	0	19	2	5
Oxoproline	0	156	72	51
Pipecolic acid	0	21	5	5
GABA	0	37	12	13
Pentosa	0	66	8	18
Ramnose	0	4	1	1
Fructose	50	2564	386	636
Mannose + Galactose	146	3698	1279	862
Glucose	262	5549	1902	1259
Glucose derivatives	57	532	221	154
Sorbose	19	297	57	70
Sucrose	275	6086	2851	1765
Rutinose + rutinose derivatives	0	58	18	18
Raffinose	0	144	15	40
Ethanolamine	0	97	18	25
Glycerol	80	505	220	117
Glycerol-3-phosphate	19	114	62	26
Arabinitol	5	44	18	9
Sorbitol	0	27	13	7
Myo-inositol	485	3375	2188	955
Methyl-inositol	831	7836	3428	2041
Ononitol	0	76	22	23
Chiro-inositol	11	686	284	219
Allo-inositol*			41	
Desoxyglucitol*			4	
Pelargonic acid (C9:0)*			2	
Undecylic acid (C11:0)	0	25	5	9
Tridecylic acid (C13:0)	0	32	6	11
Palmitic acid (C16:0)	204	859	462	179
Stearic acid (C18:0)	83	322	180	82
Arachidic acid (C20:0)*			10	
Behenic acid (C22:0)	0	26	3	7
Lignoceric acid (C24:0)	0	11	6	4
Oleic acid (C18:1)	0	248	62	58
Linoleic acid (C18:2)	24	267	127	56

Linolenic acid (C18:3)	64	469	277	108
MAG 1-C16:0	40	614	181	142
MAG 1-C18:0	8	4748	1298	1095
MAG 2-C18:2	0	5	1	2
Me C18:3	0	157	47	46
OH20:0*			10	
OH26:0	0	5	1	2
OH28:0*			3	
Isofucosterol*			10	
Campesterol	0	28	14	7
Stigmasterol	54	206	117	39
β-Sitosterol	74	237	172	49
Pyrogallol	0	4	0.4	1
Adenosine	0	43	11	14
Urea	23	236	65	54
Uridin	0	8	1	3

*– the metabolite was identified in only one sprout

Table S2. The content of metabolite compounds in the light-exposed sprouts (LS), ppm (mg/kg).

Metabolite compound	Minimum	Maximum	Mean	Standard Error
Oxalate	3	23	9	6
Lactic acid	23	106	57	20
3-Hydroxypropionic acid	2	12	5	3
Pyruvic acid	9	26	17	5
Succinic acid	29	79	47	13
Malic acid	246	1028	627	252
Fumaric acid	2	12	4	3
Tartaric acid	13	98	42	23
Citric acid	69	984	441	315
Glyceric acid	4	23	8	4
Erythronic acid	0	19	6	6
Erythrono-1.4-lactone+threono-1.4-lacton	0	6	1	2
Ribonic acid	0	41	22	10
Gluconic acid	5	39	17	11
Saccharic acid	0	38	17	14
Nicotinic acid	8	50	24	12
Shikimic acid	6	85	41	29
Quinic acid	0	6	2	3
Caffeic acid	11	95	45	26
4-Hydroxycinnamic acid	0	40	13	13
Benzoic acid	2	56	18	16
2.3-dihydrobenzoic acid	0	22	2	7
Azelaic acid*			42	
Citraconic acid	0	9	1	3

Maleic acid	0	3	1	1
Methylmalonic acid*			10	
Phosphoric acid	1084	3368	1710	719
Phosphate + methyl-phosphate	66	388	200	97
Valine	144	707	384	176
Leucine + Isoleucine	93	484	256	112
Methionine	4	62	27	23
Threonine	32	124	74	28
Phenylalanine	50	1054	464	348
Tryptophan	0	104	28	27
Lysine + Histidine	288	996	586	221
Arginine + Citrulline	68	347	145	79
α-Alanine	28	108	66	22
Glycine	5	154	44	44
Proline	7	25	18	6
Serine	28	92	50	20
Tyrosine	9	552	135	177
Aspartic acid	161	857	482	224
Asparagine	81	3894	2025	1079
Glutamic acid	381	979	543	161
Glutamine	20	128	71	31
Ornithine	72	870	428	275
Ornithine lactam	196	2254	885	648
β-Alanine	0	23	9	6
Hydroxyproline	0	33	4	9
Oxoproline	0	148	53	45
Pipecolic acid	0	10	4	3
5-Hydroxypipecolic acid	0	2	0.2	0.5
GABA	25	142	77	33
b-phenyl-a-alanine	0	20	5	8
Pentosa	0	8	2	3
Ramnose	0	9	2	3
Fructose	6	40	26	8
Mannose + Galactose	173	755	471	194
Glucose	214	994	652	264
Glucose derivatives	64	432	251	135
Sorbose	10	83	40	24
Sucrose	102	1136	506	272
Rutinose + rutinose derivatives	0	356	89	104
Raffinose	0	111	17	34
Ethanolamine	5	59	23	14
Glycerol	0	169	85	51
Glycerol-3-phosphate	13	82	36	21
Arabinitol	18	53	33	13
Sorbitol	17	74	35	20
Myo-inositol	11	236	100	69

Methyl-inositol	2897	10633	5465	2269
Ononitol	18	322	110	105
Chiro-inositol	101	849	422	229
Allo-inositol	0	24	2	7
Desoxyglucitol	0	25	14	5
Galactinols	0	100	17	27
Phytol	4	28	15	6
α-tocopherol	0	3	0.5	1.1
Pelargonic acid (C9:0)	0	2	0.2	0.7
Undecylic acid (C11:0)	0	9	1	3
Palmitic acid (C16:0)	174	423	297	73
Stearic acid (C18:0)	50	166	94	29
Arachidic acid (C20:0)	0	5	1	2
Behenic acid (C22:0)	0	21	5	8
Lignoceric acid (C24:0)	3	9	6	2
Cerotic acid (C26:0)	0	6	1	2
Oleic acid (C18:1)	0	104	20	32
Linoleic acid (C18:2)	0	95	49	34
Linolenic acid	112	418	262	111
MAG 1-C16:0	5	317	147	123
MAG 1-C18:0	1	1675	503	515
Me C18:3	0	152	20	46
OH18:0	0	6	1	2
OH22:0*			4	
OH24:0	0	6	1	2
OH26:0	0	13	3	4
OH28:0	0	16	6	4
Sterol 486	0	6	1	2
Campesterol	0	29	12	9
Stigmasterol	83	250	168	64
β-Sitosterol	13	114	56	36
Pyrogallol	0	9	4	3
Adenosine	0	5	1	2
Antirrinoside	0	5	0.7	1.7
Urea	48	670	292	181

*– the metabolite was identified in only one sprout

Table S3. The relative content of some metabolites with reference to the total content of the corresponding groups of compounds in the dark-exposed sprouts (DS), %.

**Metabolite compound**	**Minimum**	**Maximum**	**Mean**	**Standard Error**
Organic acids				
Oxalate	0.84	4.69	2.66	1.07
Lactic acid	3.95	16.65	7.11	3.02
3-Hydroxypropionic acid	0.30	1.00	0.54	0.19

Pyruvic acid	0.64	2.46	1.49	0.53
Succinic acid	4.56	7.93	6.30	0.98
Malic acid	19.28	42.59	30.13	6.35
Fumaric acid	0.24	2.28	0.52	0.49
Tartaric acid	1.38	7.51	4.06	1.52
Citric acid	0.94	11.46	5.27	3.37
Glyceric acid	1.87	4.29	3.35	0.70
Methyl-glyceric acid	0.00	0.88	0.20	0.34
Threonic acid	3.75	16.30	9.75	3.55
Erythronic acid	0.00	1.53	0.63	0.58
Erythrono-1.4-lactone+threono-1.4-lacton	0.41	1.21	0.75	0.28
Ribonic acid	0.31	3.38	2.01	0.67
Gluconic acid	1.45	18.79	9.42	5.42
Gluconic-6-phosphate acid	0.00	1.08	0.31	0.33
2-Ketogluconic acid*			0.34	
Saccharic acid	0.62	2.79	1.65	0.74
Nicotinic acid	0.47	3.21	1.34	0.77
Shikimic acid	0.48	4.92	1.90	1.06
Quinic acid	0.00	14.77	1.88	4.22
Caffeic acid	1.47	13.47	5.32	2.68
4-Hydroxycinnamic acid	0.53	5.98	2.54	1.31
Benzoic acid	0.21	0.79	0.43	0.17
2.3-dihydrobenzoic acid	0.00	0.09	0.01	0.02
Dehydroabietic acid*			1.86	
Azelaic acid	0.00	1.37	0.26	0.45
Mesoxalic acid*			0.16	
Maleic acid*			0.28	
Free amino acids				
Valine	6.67	22.69	15.39	4.51
Leucine + Isoleucine	4.25	15.83	10.97	3.49
Methionine*			0.26	
Threonine	0.00	1.79	1.07	0.39
Phenylalanine	0.75	2.83	1.42	0.58
Tryptophan	0.00	0.73	0.14	0.24
Lysine + Histidine	2.41	17.53	5.60	3.88
Arginine + Citrulline	0.00	2.18	0.89	0.69
α-Alanine	1.54	11.00	3.94	2.45
Glycine	0.01	0.66	0.41	0.18
Proline	1.04	7.82	3.04	1.78
Serine	1.04	2.99	2.27	0.53
Tyrosine	0.00	1.33	0.27	0.43
Aspartic acid	8.15	28.01	16.84	5.28
Asparagine	5.16	29.33	12.69	7.02
Glutamic acid	7.60	19.26	11.63	3.22
Glutamine	0.00	4.34	0.95	1.25
Ornithine	0.00	7.76	1.36	1.96

Ornithine lactam	0.00	16.09	6.98	4.69
β-Alanine	0.00	0.36	0.12	0.14
Hydroxyproline	0.00	0.87	0.06	0.22
Oxoproline	0.00	7.57	3.17	2.27
Pipecolic acid	0.00	0.94	0.24	0.23
GABA	0.00	1.95	0.53	0.60
Saccharides				
Pentosa	0.00	0.41	0.07	0.12
Ramnose	0.00	0.10	0.01	0.03
Fructose	1.72	19.39	5.60	4.92
Mannose + Galactose	12.21	25.06	18.94	4.18
Glucose + Glucose derivatives	18.87	44.37	33.63	6.92
Sorbose	0.34	2.24	0.94	0.60
Sucrose	25.65	55.70	40.28	9.95
Rutinose + rutinose derivatives	0.00	1.30	0.35	0.41
Raffinose	0.00	1.54	0.18	0.47
Total monosaccharides	43.97	74.09	59.20	9.80
Total disaccharides	25.91	56.03	40.63	9.81
Total trisaccharides	0.00	1.54	0.18	0.47
Alcohols				
Ethanolamine	0.00	1.32	0.26	0.34
Glycerol	1.22	13.73	4.67	3.54
Glycerol-3-phosphate	0.42	6.77	1.40	1.60
Arabinitol	0.14	0.77	0.35	0.19
Sorbitol	0.00	0.28	0.20	0.07
Myo-inositol	18.10	49.43	34.74	8.33
Methyl-inositol	40.63	75.78	54.37	9.55
Ononitol	0.00	0.65	0.27	0.21
Chiro-inositol	0.68	8.44	3.72	2.17
Allo-inositol*			0.43	
Desoxyglucitol*			0.04	
Fatty acids				
Pelargonic acid (C9:0)			0.21	
Undecylic acid (C11:0)	0.00	1.41	0.32	0.55
Tridecylic acid (C13:0)	0.00	1.48	0.32	0.58
Palmitic acid (C16:0)	31.64	61.76	39.73	7.95
Stearic acid (C18:0)	11.05	23.61	14.92	3.62
Arachidic acid (C20:0)			0.92	
Behenic acid (C22:0)	0.00	1.31	0.19	0.44
Lignoceric acid (C24:0)	0.00	0.98	0.46	0.32
Oleic acid (C18:1)	0.00	11.64	4.99	2.68
Linoleic acid (C18:2)	3.98	13.16	10.81	2.68
Linolenic acid (C18:3)	10.48	36.25	28.04	7.41
Total saturated fatty acids	45.01	82.70	56.17	10.73
Total unsaturated fatty acids	17.30	54.99	43.83	10.73
Phytosterols				

Campesterol	0.00	9.13	4.47	2.18
Stigmasterol	28.99	49.14	38.44	4.77
β-Sitosterol	50.08	61.88	56.87	3.24
Isofucosterol			3.29	
Phenolic acids				
Caffeic acid	48.67	78.52	63.68	8.09
4-Hydroxycinnamic acid	16.53	45.23	30.38	7.62
Benzoic acid + 2.3-dihydrobenzoic acid	2.34	10.25	5.77	2.02

*– the metabolite was identified in only one sprout

Table S4. The relative content of some metabolites with reference to the total content of the corresponding groups of compounds in the light–exposed sprouts (LS), %.

**Metabolite compound**	**Minimum**	**Maximum**	**Mean**	**Standard Error**
Organic acids				
Oxalate	0.17	1.21	0.65	0.33
Lactic acid	1.26	10.37	4.85	3.16
3-Hydroxypropionic acid	0.15	1.04	0.42	0.28
Pyruvic acid	0.73	1.97	1.23	0.43
Succinic acid	1.49	9.03	3.84	2.49
Malic acid	33.53	60.68	45.06	7.72
Fumaric acid	0.17	0.59	0.29	0.10
Tartaric acid	1.44	5.28	2.84	1.13
Citric acid	10.42	43.05	25.92	11.14
Glyceric acid	0.35	2.51	0.66	0.55
Erythronic acid	0.00	0.99	0.42	0.33
Erythrono-1.4-lactone+threono-1.4-lacton	0.00	0.62	0.07	0.18
Ribonic acid	0.00	2.31	1.45	0.52
Gluconic acid	0.54	2.38	1.24	0.52
Saccharic acid	0.00	2.81	1.21	0.76
Nicotinic acid	0.51	7.22	2.07	1.96
Shikimic acid	0.78	5.57	2.66	1.44
Quinic acid	0.00	0.75	0.19	0.30
Caffeic acid	1.68	7.72	2.96	1.52
4-Hydroxycinnamic acid	0.00	1.94	0.56	0.69
Benzoic acid	0.00	2.44	0.75	0.77
2.3-dihydrobenzoic acid	0.00	1.20	0.13	0.35
Azelaic acid			5.20	
Citraconic acid	0.00	1.11	0.11	0.30
Maleic acid	0.00	0.15	0.03	0.05
Methylmalonic acid			0.52	
Free amino acids				
Valine	2.04	7.52	5.54	1.41
Leucine + Isoleucine	1.50	5.64	3.75	1.00
Methionine	0.07	1.64	0.49	0.53
Threonine	0.79	1.52	1.09	0.21

Phenylalanine	1.10	10.70	5.97	3.23
Tryptophan	0.00	1.28	0.41	0.37
Lysine + Histidine	6.33	12.25	8.76	1.68
Arginine + Citrulline	1.12	3.59	2.10	0.68
α-Alanine	0.32	1.91	1.05	0.41
Glycine	0.14	1.76	0.60	0.47
Proline	0.08	0.47	0.28	0.10
Serine	0.49	1.28	0.75	0.25
Tyrosine	0.25	5.83	1.84	2.26
Aspartic acid	1.84	18.12	8.14	4.89
Asparagine	1.40	41.04	29.08	11.20
Glutamic acid	3.95	13.71	8.28	2.82
Glutamine	0.31	2.06	1.10	0.46
Ornithine	2.18	9.48	5.68	2.50
Ornithine lactam	4.59	38.88	12.93	8.73
β-Alanine	0.00	0.28	0.12	0.07
Hydroxyproline	0.00	0.41	0.05	0.12
Oxoproline	0.00	2.55	0.73	0.67
Pipecolic acid	0.00	0.13	0.07	0.05
5-Hydroxypipecolic acid			0.02	
GABA	0.55	1.87	1.14	0.38
b-phenyl-a-alanine	0.00	0.20	0.06	0.09
Saccharides				
Pentosa	0.00	0.27	0.07	0.10
Ramnose	0.00	0.30	0.08	0.09
Fructose	0.88	4.03	1.38	0.82
Mannose + Galactose	15.65	29.40	22.92	3.68
Glucose + Glucose derivatives	29.63	55.54	43.79	6.94
Sorbose	0.75	3.39	1.94	0.75
Sucrose	8.73	51.69	25.53	10.93
Rutinose + rutinose derivatives	0.00	14.18	3.69	4.03
Raffinose	0.00	3.53	0.59	1.10
Total monosaccharides	47.67	86.93	70.19	10.45
Total disaccharides	13.01	51.82	29.22	10.07
Total trisaccharides	0.00	3.53	0.59	1.10
Alcohols				
Ethanolamine	0.07	1.10	0.44	0.30
Glycerol	0.00	4.05	1.51	0.99
Glycerol-3-phosphate	0.29	0.98	0.57	0.21
Arabinitol	0.31	1.26	0.55	0.25
Sorbitol	0.32	0.89	0.54	0.15
Myo-inositol	0.21	5.22	1.59	1.28
Methyl-inositol	79.63	94.18	85.75	4.09
Ononitol	0.53	3.66	1.48	1.06
Chiro-inositol	0.90	10.24	6.70	2.08
Allo-inositol*			0.22	

Desoxyglucitol	0.00	0.40	0.22	0.11
Phytol	0.06	0.62	0.26	0.15
Fatty acids				
Pelargonic acid (C9:0)	0.00	0.36	0.04	0.10
Undecylic acid (C11:0)	0.00	1.43	0.15	0.41
Palmitic acid (C16:0)	33.26	48.99	40.17	4.78
Stearic acid (C18:0)	0.00	18.34	11.43	4.10
Arachidic acid (C20:0)	0.00	0.47	0.13	0.18
Behenic acid (C22:0)	0.00	4.83	0.99	1.67
Lignoceric acid (C24:0)	0.52	1.68	0.86	0.29
Cerotic acid (C26:0)	0.00	1.24	0.53	0.41
Oleic acid (C18:1)	0.00	12.07	2.65	3.67
Linoleic acid (C18:2)	0.00	10.47	6.09	3.09
Linolenic acid	24.84	45.71	36.16	6.26
Phytosterols				
Sterol 486	0.00	1.63	0.37	0.65
Campesterol	0.00	8.51	4.26	2.40
Stigmasterol	56.74	86.25	74.03	8.69
β-Sitosterol	13.68	37.99	21.33	7.18
Phenolic acids				
Caffeic acid	31.41	100.00	66.00	21.32
4-Hydroxycinnamic acid**	0.00	30.27	15.47	12.14
Benzoic acid + 2.3-dihydrobenzoic acid	0.00	46.79	16.21	14.98

*– the metabolite was identified in only one sprout

**– the value is calculated only for LS of accessions k-14408 and k-14016

Table S5. The results of one-way ANOVA (analysis of variance) to identify associations between the metabolite content variability in mung bean sprouts and the conditions of their germination.

Effect	Degr. of Freedom	SS	MS	F	p	SS	MS	F	p	SS	MS	F	p
		lactic acid				oxalate				succinic acid			
**Conditions**	1	23304.1	23304.1	8.6	0.01	8147.7	8147.7	22.6	0.01×10^{-2}	20003.2	20003.2	40.4	0.01×10^{-4}
**Error**	26	70858.8	2725.3			9385.0	361.0			12857.7	494.5		
**Total**	27	94162.9				17532.8				32860.9			
**η^2,%**		24.7				46.5				60.9			
		pyruvic acid				ribonic acid				phosphate + methyl phosphate			
**Conditions**	1	269.2	269.2	5.2	0.03	641.3	641.3	4.7	0.04	39748.0	39748.0	4.5	0.04
**Error**	26	1349.6	51.9			3511.7	135.1			227519.9	8750.8		
**Total**	27	1618.8				4153.0				267268.0			
**η^2,%**		16.6				15.4				14.9			
		citric acid				tartaric acid				3-hydroxypropionic acid			
**Conditions**	1	835552.3	835552.3	15.6	0.01×10^{-1}	4292.7	4292.7	7.0	0.01	51.4	51.4	9.2	0.01
**Error**	26	1394537.3	53636.0			15962.0	613.9			144.9	5.6		
**Total**	27	2230089.5				20254.6				196.3			
**η^2,%**		37.5				21.2				26.2			
		4-hydroxycinnamic acid				glyceric acid				methylglyceric acid			
**Conditions**	1	5597.2	5597.2	8.8	0.01	13535.1	13535.1	141.3	0.01×10^{-9}	79.2	79.2	4.5	0.04
**Error**	26	16473.6	633.6			2490.1	95.8			460.7	17.7		
**Total**	27	22070.8				16025.3				539.9			
**η^2,%**		25.4				84.5				14.7			

Effect	Degr. of Freedom	SS	MS	F	p	SS	MS	F	p	SS	MS	F	p
		threonic acid				threono-lactone + erythrono-lactone				gluconic acid			
**Conditions**	1	172084.3	172084.3	93.0	0.04×10^{-8}	877.3	877.3	54.0	0.01×10^{-5}	146714.8	146714.8	23.2	0.01×10^{-2}
**Error**	26	48120.4	1850.8			422.4	16.2			164686.1	6334.1		
**Total**	27	220204.7				1299.7				311400.9			
**η^2,%**		78.1				67.5				47.1			
		gluconic-6-phosphate				methionine				threonine			
**Conditions**	1	190.8	190.8	19.7	0.01×10^{-2}	4885.1	4885.1	18.2	0.02×10^{-2}	17492.6	17492.6	34.2	0.04×10^{-4}
**Error**	26	251.8	9.7			6975.1	268.3			13301.6	511.6		
**Total**	27	442.6				11860.2				30794.2			
**η^2,%**		43.1				41.2				56.8			
		phenylalanine				tryptophan				glycine			
**Conditions**	1	1295678.6	1295678.6	21.3	0.01×10^{-2}	3793.1	3793.1	9.7	0.04×10^{-1}	8523.9	8523.9	8.6	0.01
**Error**	26	1582962.1	60883.2			10166.3	391.0			25636.0	986.0		
**Total**	27	2878640.7				13959.4				34159.9			
**η^2,%**		45.0				27.2				25.0			
		tyrosine				histidine + lysine				asparagine			
**Conditions**	1	112305.0	112305.0	7.1	0.01	1653298.9	1653298.9	62.5	0.02×10^{-6}	21067674.4	21067674.4	34.6	0.03×10^{-4}
**Error**	26	412569.3	15868.0			688165.4	26467.9			15834159.1	609006.1		
**Total**	27	524874.3				2341464.3				36901833.5			
**η^2,%**		21.4				70.6				57.1			
		aspartate				b-alanine				ornithine lactam			
**Conditions**	1	173274.7	173274.7	5.0	0.03	207.4	207.4	7.5	0.01	3451557.9	3451557.9	15.0	0.01×10^{-1}
**Error**	26	899660.9	34602.3			718.0	27.6			5989713.3	230373.6		
**Total**	27	1072935.6				925.4				9441271.2			
**η^2,%**		16.1				22.4				36.6			

Effect	Degr. of Freedom	SS	MS	F	p	SS	MS	F	p	SS	MS	F	p
		glutamate				glutamine				proline			
**Conditions**	1	696040.1	696040.1	36.4	0.02×10^{-4}	19760.0	19760.0	23.9	0.04×10^{-3}	11952.2	11952.2	13.3	0.01×10^{-1}
**Error**	26	497461.5	19133.1			21478.3	826.1			23294.5	895.9		
**Total**	27	1193501.6				41238.3				35246.7			
**η^2,%**		58.3				47.9				33.9			
		citrulline – arginine				GABA				ornithine			
**Conditions**	1	110524.4	110524.4	34.1	0.04×10^{-4}	29192.8	29192.8	45.9	0.03×10^{-4}	1069021.0	1069021.0	26.8	0.02×10^{-3}
**Error**	26	84393.7	3245.9			16545.0	636.3			1037576.8	39906.8		
**Total**	27	194918.0				45737.8				2106597.7			
**η^2,%**		56.7				63.8				50.7			
		b-phenyl-a-alanine				glycerol				arabinitol			
**Conditions**	1	193.0	193.0	6.2	0.02	126561.3	126561.3	15.6	0.01×10^{-1}	1711.4	1711.4	13.0	0.01×10^{-1}
**Error**	26	803.3	30.9			211092.0	8118.9			3421.6	131.6		
**Total**	27	996.3				337653.3				5133.0			
**η^2,%**		19.4				37.5				33.3			
		threitol + erythritol				glycerol-3-phosphate				methyl-inositol			
**Conditions**	1	187.7	187.7	9.2	0.01	4533.1	4533.1	8.3	0.01	29051842.5	29051842.5	6.2	0.02
**Error**	26	532.5	20.5			14122.6	543.2			121077779.1	4656837.7		
**Total**	27	720.2				18655.7				150129621.6			
**η^2,%**		26.1				24.3				19.4			
		desoxyglucitol				sorbitol				ononitol			
**Conditions**	1	1259.8	1259.8	83.0	0.01×10^{-7}	3167.3	3167.3	14.3	0.01×10^{-1}	54508.1	54508.1	9.4	0.01
**Error**	26	394.5	15.2			5766.3	221.8			151029.6	5808.8		
**Total**	27	1654.3				8933.6				205537.6			
**η^2,%**		76.2				35.5				26.5			

Effect	Degr. of Freedom	SS	MS	F	p	SS	MS	F	p	SS	MS	F	p
		myo-inositol				galactinols				phytol			
**Conditions**	1	30507486.5	30507486.5	66.5	0.01×10^{-6}	2103.9	2103.9	5.9	0.02	1485.9	1485.9	80.0	0.02×10^{-7}
**Error**	26	11924789.4	458645.7			9230.8	355.0			482.8	18.6		
**Total**	27	42432275.9				11334.7				1968.8			
**η^2,%**		71.9				18.6				75.5			
		sucrose				fructose				rutinose and derivatives			
**Conditions**	1	38468142.7	38468142.7	24.1	0.04×10^{-3}	910427.8	910427.8	4.5	0.04	36152.4	36152.4	6.5	0.02
**Error**	26	41467182.9	1594891.7			5265822.3	202531.6			144059.8	5540.8		
**Total**	27	79935325.6				6176250.1				180212.2			
**η^2,%**		48.1				14.7				20.1			
		mannose + galactose				glucose							
**Conditions**	1	4566223.1	4566223.1	11.7	0.02×10^{-1}	10950048.3	10950048.3	13.2	0.01×10^{-1}				
**Error**	26	10140808.0	390031.1			21524055.1	827848.3						
**Total**	27	14707031.1				32474103.4							
**η^2,%**		31.0				33.7							
		stigmasterol				β-sitosterol				palmitic acid (C16:0)			
**Conditions**	1	18487.8	18487.8	6.6	0.02	95272.2	95272.2	51.3	0.01×10^{-5}	190897.0	190897.0	10.2	0.04×10^{-1}
**Error**	26	73182.4	2814.7			48287.1	1857.2			486916.5	18727.6		
**Total**	27	91670.1				143559.3				677813.5			
**η^2,%**		20.2				66.4				28.2			
		stearic acid (C18:0)				arachidic acid (C20:0)				oleic acid (C18:1)			
**Conditions**	1	61452.6	61452.6	14.9	0.01×10^{-1}	12.7	12.7	8.5	0.01	12304.2	12304.2	5.6	0.03
**Error**	26	107259.8	4125.4			39.0	1.5			57532.0	2212.8		
**Total**	27	168712.4				51.8				69836.1			
**η^2,%**		36.4				24.6				17.6			

Effect	Degr. of Freedom	SS	MS	F	p	SS	MS	F	p	SS	MS	F	p
		linoleic acid (C18:2)				OH28:0				acylglycerols			
**Conditions**	1	42312.6	42312.6	19.7	0.01×10^{-2}	224.1	224.1	21.6	0.01×10^{-2}	4810695.4	4810695.4	5.1	0.03
**Error**	26	55928.2	2151.1			269.9	10.4			24548107.4	944158.0		
**Total**	27	98240.8				494.1				29358802.7			
**η^2,%**		43.1				45.4				16.4			
		pyrogallol				urea				adenosine			
**Conditions**	1	106.5	106.5	16.7	0.04×10^{-2}	360308.1	360308.1	20.1	0.01×10^{-2}	696.1	696.1	7.2	0.01
**Error**	26	166.1	6.4			465672.0	17910.5			2526.3	97.2		
**Total**	27	272.6				825980.1				3222.4			
**η^2,%**		39.1				43.6				21.6			

SS – sum of squares; MS – mean squares; F – Fisher criterion value; p – significance level; η^2, % – effect size, percentage; Total – total variability; Error – residual variability.

Table S6. The results of one-way ANOVA (analysis of variance) to identify associations between the metabolite content variability in mung bean sprouts and the accessions (genotype).

Effect	Degr. of Freedom	SS	MS	F	p	SS	MS	F	p	SS	MS	F	p
		Citric acid				Nicotinic acid				4-hydroxycinnamic acid			
**Accessions**	2	483964.1	241982.0	3.5	0.04	929.7	464.9	4.3	0.02	4973.1	2486.6	3.6	0.04
**Error**	25	1746125.4	69845.0			2687.2	107.5			17097.7	683.9		
**Total**	27	2230089.5				3616.9				22070.8			
**η^2,%**		21.7				25.7				22.5			
		Ribonic acid				Erythronic acid				Valine			

Effect	Degr. of Freedom	SS	MS	F	p	SS	MS	F	p	SS	MS	F	p
**Accessions**	2	1212.6	606.3	5.2	0.01	520.7	260.4	5.4	0.01	481966.3	240983.1	6.7	0.004
**Error**	25	2940.4	117.6			1208.9	48.4			895389.6	35815.6		
**Total**	27	4153.0				1729.6				1377355.9			
**η²,%**		29.2				15.4				35.0			
		Leucine + isoleucine				Tryptophan				Serine			
**Accessions**	2	236053.4	118026.7	6.2	0.006	3648.6	1824.3	4.4	0.02	6628.1	3314.1	7.7	0.002
**Error**	25	478700.8	19148.0			10310.8	412.4			10724.5	429.0		
**Total**	27	714754.2				13959.4				17352.7			
**η²,%**		33.0				26.1				38.2			
		Tyrosine				Oxoproline				Sorbitol			
**Accessions**	2	130994.8	65497.4	4.2	0.02	33315.7	16657.9	13.9	0.00008	2959.9	1480.0	6.2	0.006
**Error**	25	393879.5	15755.2			29908.6	1196.3			5973.7	238.9		
**Total**	27	524874.3				63224.4				8933.6			
**η²,%**		25.0				52.7				33.1			
		Methyl-inositol				Ononitol				Chiro-inositol			
**Accessions**	2	52544418.0	26272209.0	6.7	0.004	67733.5	33866.8	6.1	0.006	465409.9	232705.0	6.0	0.007
**Error**	25	97585203.6	3903408.1			137804.1	5512.2			975964.6	39038.6		
**Total**	27	150129621.6				205537.6				1441374.5			
**η²,%**		35.0				33.0				32.3			
		Rhamnose				Glucose derivatives				Rutinose and derivatives			
**Accessions**	2	37.9	19.0	5.6	0.009	307246.3	153623.1	16.0	0.00009	56495.1	28247.6	5.7	0.009
**Error**	25	84.3	3.4			240730.2	9629.2			123717.1	4948.7		
**Total**	27	122.2				547976.5				180212.2			
**η²,%**		31.0				56.1				31.3			
		Campesterol				Stigmasterol				C20:0			

Effect	Degr. of Freedom	SS	MS	F	p	SS	MS	F	p	SS	MS	F	p
**Accessions**	2	412.4	206.2	3.8	0.04	34352.4	17176.2	7.5	0.002	15.5	7.7	5.3	0.01
**Error**	25	1345.4	53.8			57317.8	2292.7			36.3	1.5		
**Total**	27	1757.9				91670.1				51.8			
**η^2,%**		23.5				37.5				29.9			
		C18:3				Acylglycerols				Adenosine			
**Accessions**	2	91561.8	45780.9	5.1	0.01	8183609.2	4091804.6	4.8	0.02	815.4	407.7	4.2	0.02
**Error**	25	222470.8	8898.8			21175193.5	847007.7			2407.1	96.3		
**Total**	27	314032.6				29358802.7				3222.4			
**η^2,%**	25	29.2				27.9				25.3			

SS – sum of squares; MS – mean squares; F – Fisher criterion value; p – significance level; η^2, % – effect size, percentage; Total – total variability; Error – residual variability.

Table S7. Factorial Anova results to identify relationships between the variability of metabolite content in mungbean sprouts, the accession (genotype), the sprout germination conditions and the interaction of conditions*accessions (genotypes)

Effect	Df	SS	MS	F	p	SS	MS	F	p	SS	MS	F	p
		Citric acid				4-hydroxycinnamic acid				Ribonic acid			
**Accessions**	2	**483964.1**	**241982.0**	**20.5**	**0.09×10^{-4}**	**4973.15**	**2486.57**	**5.02803**	**0.02**	**1212.6**	**606.3**	**6.1**	**0.008**
**Conditions**	1	**840687.6**	**840687.6**	**71.2**	**0.02×10^{-5}**	**5331.16**	**5331.16**	**10.77997**	**0.003**	**592.4**	**592.4**	**5.9**	**0.02**
**Conditions*Accessions**	2	**650765.8**	**325382.9**	**27.5**	**0.01×10^{-3}**	620.49	310.24	0.62734	0.54	110.5	55.24	0.6	0.58
**Error**	22	259807.4	11809.4			10879.9	494.5			2188.6	99.5		
**Total**	27	2230090.5				22070.8				4153.0			

Effect	Df	SS	MS	F	p	SS	MS	F	p	SS	MS	F	p
η^2A, %		**21.7**				**22.5**				**29.2**			
η^2C, %		**37.7**				**24.2**				**14.3**			
η^2 C*A,%		**29.2**				**2.8**				2.7			
		Erythronic acid				Tryptophan				Tyrosine			
**Accessions**	2	**520.7**	**260.4**	**5.1**	**0.02**	**3648.6**	**1824.3**	**7.4**	**0.004**	**130994.8**	**65497.4**	**7.8**	**0.003**
**Conditions**	1	**78.6**	**78.6**	**1.5**	**0.23**	**3783.6**	**3783.6**	**15.3**	**0.001**	**98685.3**	**98685.3**	**11.8**	**0.002**
**Conditions*Accessions**	2	2.1	1.0	0.0	0.98	1078.1	539.1	2.2	0.14	**96876.4**	**48438.2**	**5.8**	**0.01**
**Error**	22	1129.0	51.3			5439.5	247.3			184698.1	8395.4		
**Total**	27	1729.6				13959.4				524874.3			
η^2A, %		**30.1**				**26.1**				**25.0**			
η^2C, %		**4.5**				**27.1**				**18.8**			
η^2 C*A,%		0.1				7.7				**18.5**			
		Sorbitol				Methyl-inositol				Ononitol			
**Accessions**	2	**2959.9**	**1480.0**	**15.6**	**0.01×10^{-2}**	**52544418.0**	**26272209.0**	**8.6**	**0.002**	**67733.5**	**33866.8**	**14.1**	**0.01×10^{-2}**
**Conditions**	1	**3053.1**	**3053.1**	**32.1**	**0.01×10^{-3}**	**30106519.1**	**30106519.1**	**9.9**	**0.005**	**51860.3**	**51860.3**	**21.5**	**0.01×10^{-2}**
**Conditions*Accessions**	2	**714.5**	**357.3**	**3.8**	**0.04**	1575428.6	787714.3	0.3	0.77	**30333.6**	**15166.8**	**6.3**	**0.007**
**Error**	22	2091.8	95.1			66957932.5	3043542.4			52962.4	2407.4		
**Total**	27	8933.6				150129621.6				205537.6			
η^2A, %		**33.1**				**35.0**				**33.0**			
η^2C, %		**34.2**				**20.1**				**25.2**			
η^2 C*A,%		**8.0**				1.0				**14.8**			
		Rutinose and derivatives				Stigmasterol				Acylglycerols			

Effect	Df	SS	MS	F	p	SS	MS	F	p	SS	MS	F	p
**Accessions**	2	**56495.1**	**28247.6**	**16.8**	**0.04×10^{-3}**	**34352.4**	**17176.2**	**16.7**	**0.04×10^{-3}**	**8183609.2**	**4091804.6**	**6.3**	**0.01**
**Conditions**	1	**44516.6**	**44516.6**	**26.5**	**0.04×10^{-3}**	**20124.7**	**20124.7**	**19.6**	**0.02×10^{-3}**	**4907770.5**	**4907770.5**	**7.5**	**0.01**
**Conditions*Accessions**	2	**50571.8**	**25285.9**	**15.0**	**0.08×10^{-3}**	**16238.8**	**8119.4**	**7.9**	**0.003**	2059111.9	1029556.0	1.6	0.23
**Error**	22	36992.9	1681.5			22591.2	1026.9			14305386.2	650244.8		
**Total**	27	180212.2				91670.1				29358802.7			
**η^2A, %**		**31.3**				**37.5**				**27.9**			
**η^2C, %**		**24.7**				**22.0**				**16.7**			
**η^2 C*A,%**		**28.1**				**17.7**				7.0			

SS – sum of squares; MS – mean squares; F – Fisher criterion value; p – significance level; df –degrees of freedom; η^2A, % – effect size (Accessions), percentage; η^2C, % – effect size (Conditions), percentage; η^2A*C, % – effect size (Accessions* Conditions), percentage; Total – total variability; Error – residual variability. Reliable data is highlighted in bold

Table S8. Factor loadings on the characters (identified compounds) of mung bean sprouts.

Characters	Factor 1	Factor 2	Factor 3
Lactic acid	0.686221	0.146976	0.001263
Pyruvic acid	0.585759	0.42328	-0.05038
Oxalic acid	0.711	0.229526	0.359304
Succinic acid	0.854075	0.252457	0.183851
Malic acid	-0.08076	0.639518	-0.58716
Shikimic acid	0.070868	0.556096	-0.69704
Citric acid	-0.63744	0.689181	-0.15812
Quinic acid	0.333169	0.278373	-0.43568
Tartaric acid	0.580958	0.440672	0.147903
Fumaric acid	0.544521	0.632771	-0.14688
3-hydroxypropionic acid	0.538392	0.443998	0.339974
Caffeic acid	0.445336	0.270756	0.266006
2.3-dihydroxybenzoic acid	-0.27578	0.297858	-0.12136
Benzoic acid	-0.38453	0.599171	0.039918
Nicotinic acid	-0.16276	0.267771	0.633251
4-hydroxycinnamic acid	0.44111	0.346902	0.512598
Maleic acid	-0.31918	0.363778	-0.07509
Azelaic acid	0.137888	-0.23363	-0.18008
Aconitic acid	-0.51079	0.529946	0.151912
Dehydroabietic acid	0.004739	-0.31875	-0.01503
Methylmalonic acid	-0.10401	-0.02375	-0.27629
Citraconic acid	-0.16011	-0.27249	-0.17179
Mesoxalic acid	0.213338	0.118137	0.376302
Saccharic acid	0.355661	0.505164	-0.00339
Glyceric acid	0.926924	0.127096	0.202776
Methylglyceric acid	0.52233	0.049249	-0.21337
Ribonic acid	0.435944	0.504105	0.348986
Erythronic acid	0.034388	0.382241	0.724504
Threonic acid	0.843159	0.153529	0.42028
Total lactones of threonic and erythronic acids	0.883638	0.179014	0.104172
Gluconic acid	0.75075	0.196993	0.331246
6-phosphogluconic acid	0.603613	0.078062	0.26874
2-ketogluconic acid	0.164774	-0.00526	0.1679
Phosphoric acid	-0.40792	0.402941	0.22775
Phosphate + methyl phosphate	0.349352	0.650846	0.260129
Valine	-0.26867	-0.10305	0.604876
Leucine + isoleucine	-0.25049	0.045866	0.577201
Methionine	-0.51798	0.056528	-0.46491
Threonine	-0.86074	0.185528	0.121663

Phenylalanine	-0.72458	-0.08761	0.129495
Tryptophan	-0.63222	0.555416	0.10731
α-alanine	-0.10044	0.513381	0.015175
Glycine	-0.60014	-0.05266	0.177716
Serine	-0.28481	0.314157	0.496309
Tyrosine	-0.62836	0.586863	0.259194
Histidine + lysine	-0.86172	0.073493	-0.02412
Aspartic acid	-0.37192	0.319679	-0.23012
Asparagine	-0.6994	-0.36926	-0.17738
Glutamic acid	-0.71081	-0.15564	-0.00668
Glutamine	-0.7773	0.289922	-0.01482
Proline	0.397112	0.065202	0.438458
Citrulline + arginine	-0.84845	0.31412	0.069105
β-alanine	-0.73194	0.194718	0.318735
GABA	-0.85581	-0.04492	0.077458
Oxoproline	0.02632	0.017276	0.667448
Hydroxyproline	-0.03245	-0.19792	-0.18859
Ornithine	-0.76452	-0.23415	0.086787
Pipecolic acid	0.029242	0.58767	-0.17101
5-hydroxypipecolic acid	-0.23014	-0.10596	0.136299
β-phenyl-α-alanine	-0.57913	0.203182	0.233977
Ornithine lactam	-0.72419	0.350991	0.261883
Ethanolamine	0.037943	0.026946	-0.08833
Glycerol	0.754902	0.222357	-0.20585
Threitol + erythritol	-0.56124	0.43559	0.064016
Arabinitol	-0.43644	0.563598	-0.21043
Glycerol-3-phosphate	0.472114	0.586442	0.131162
Deoxyglucitol	-0.8023	0.259372	-0.13423
Sorbitol	-0.68002	0.61717	0.236821
Allo-inositol	-0.01827	0.075604	0.282131
Methyl-inositol	-0.45731	0.492002	0.349789
Ononitol	-0.63065	0.645629	0.118337
Chiro-inositol	-0.46959	0.418404	0.368508
Myo-inositol	0.79087	0.090305	0.486719
Galactinols	-0.36429	0.236661	-0.39724
Phytol	-0.75454	0.120141	-0.35585
α-tocopherol	-0.48492	0.53333	0.108468
Pentoses	0.427297	0.170023	-0.07386
Rhamnose	-0.44898	0.493839	0.162112
Fructose	0.522363	0.304799	-0.38717
Mannose + galactose	0.728945	0.378065	-0.09814
Glucose	0.746559	0.357251	-0.06253
Glucose derivatives	0.037945	0.600154	-0.15994
Sorbose	0.290951	0.56109	-0.40625

Sucrose	0.835551	0.302587	0.002199
Rutinose and derivatives	-0.36063	0.226583	-0.44812
Raffinose. etc.	0.026096	0.435903	-0.45564
Campesterol	0.109844	0.665374	-0.20313
Stigmasterol	-0.31268	0.628958	-0.28188
β-sitosterol	0.759476	0.446855	0.306284
sterol 486	-0.2494	0.159912	-0.40711
Isofucosterol	0.061512	-0.00498	0.303036
C9:0	0.00777	-0.04431	0.279377
C11:0	0.440758	0.232519	-0.49224
C13:0	0.5928	0.324754	-0.42091
C16:0	0.739909	0.465344	-0.29819
C18:0	0.742555	0.351589	-0.1701
C20:0	-0.54366	0.642872	0.037129
C22:0	-0.09255	-0.13022	-0.32834
C24:0	-0.01028	0.647772	0.064665
C26:0	-0.47362	0.518132	0.097711
C18:1	0.415525	0.410939	-0.11535
C18:2	0.748781	0.421113	-0.10284
C18:3	0.216096	0.766061	-0.24911
MeC18:3	0.288735	-0.01409	0.555959
OH18:0	-0.48307	0.571344	0.24081
OH20:0	0.213338	0.118137	0.376302
OH22:0	-0.14023	0.094311	-0.24934
OH24:0	-0.44485	0.525429	0.237661
OH26:0	-0.23157	0.253894	-0.31175
OH28:0	-0.60019	0.234025	-0.36192
Acylglycerols	0.582433	0.342166	-0.24569
Pyrogallol	-0.62338	0.171726	-0.09538
Urea	-0.74231	0.016244	0.204859
Adenosine	0.464994	0.224936	-0.1643
Uridine	0.361193	0.168549	-0.40776
Antirrinoside.	-0.36919	0.400405	0.025232
**Expl.Var**	31.75218	16.24177	10.35937
**Prp.Totl**	0.273726	0.140015	0.089305

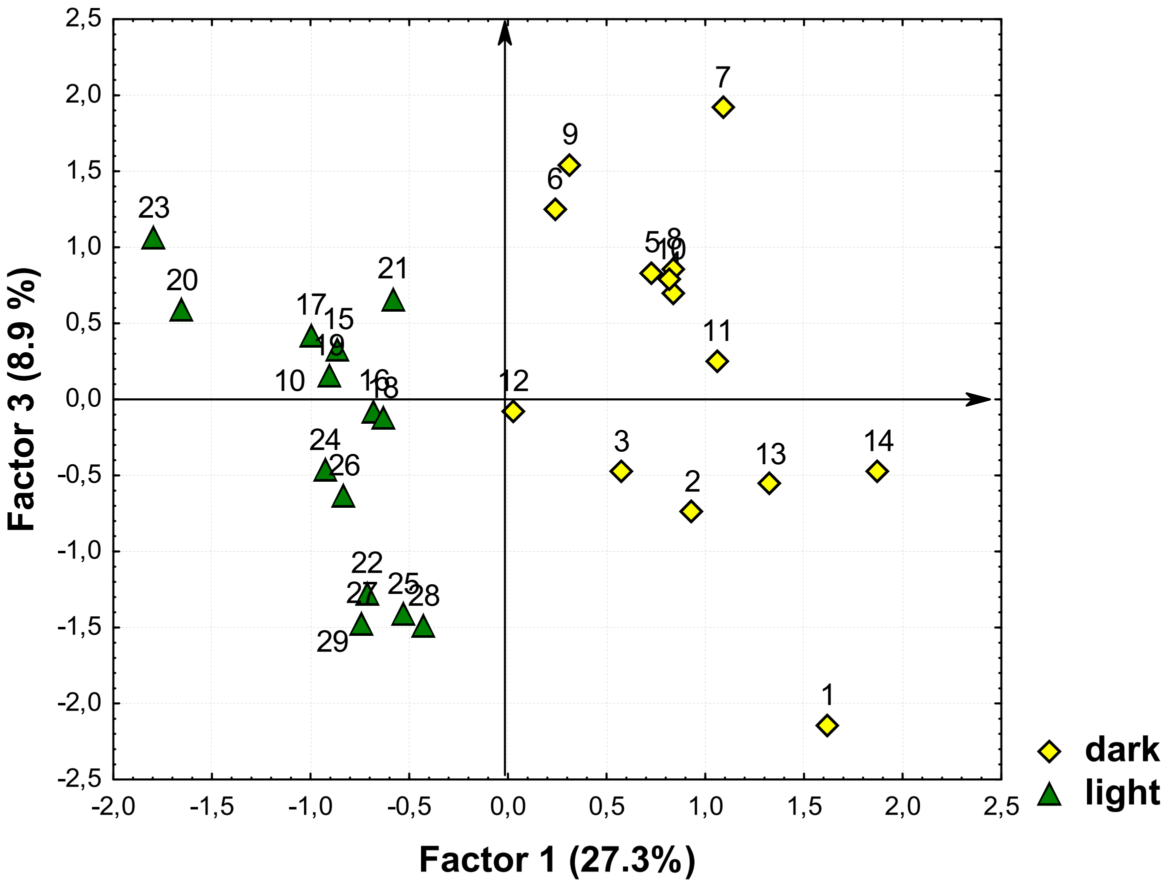

Figure S1. A scatterplot of mung bean sprouts in the space of the first and third factors, calculated according to the content of all identified compounds.

Table S9. Factor loadings on the characters (calculated as percentages of individual compounds in the total amount of each identified class of compounds (total organic acids, amino acids, saccharides, alcohols, lipids, and phytosterols).

Characters	Factor 1	Factor 2	Factor 3
Lactic acid	0.437561	0.734319	-0.18923
Pyruvic acid	0.33522	0.547552	-0.21778
Oxalic acid	0.791274	0.046994	-0.17811
Succinic acid	0.608265	0.449354	-0.46111
Malic acid	-0.70822	0.217165	0.323612
Shikimic acid	-0.30427	-0.25271	0.813118
Citric acid	-0.84496	-0.29553	0.081548
Quinic acid	0.277906	-0.11778	0.492789
Tartaric acid	0.493007	0.250759	-0.03498
Fumaric acid	0.289752	-0.09274	0.072914
3-Hydroxypropionic acid	0.254333	0.448513	-0.5914
Caffeic acid	0.4997	-0.15436	-0.07859
2.3-dihydroxybenzoic acid	-0.29672	-0.32357	0.087609

Benzoic acid	-0.38048	-0.14227	-0.08323
Nicotinic acid	-0.20189	0.435633	-0.62405
4-hydroxycinnamic acid	0.613138	-0.39058	-0.3105
Malic acid	-0.10486	-0.32826	0.064813
Azelaic acid	0.003757	0.32585	-0.0047
Aconitic acid	-0.42448	-0.27015	-0.02795
Dehydroabietic acid	0.114083	0.054431	-0.09964
Methylmalonic acid	-0.16211	-0.0392	0.189548
Citraconic acid	-0.18924	0.260183	0.024729
Mesoxalic acid	0.176994	-0.26894	-0.21735
Saccharic acid	0.257795	0.099342	0.204198
Glyceric acid	0.899465	0.09659	-0.17026
Methylglyceric acid	0.421297	0.459812	0.378895
Ribonic acid	0.454008	-0.04669	-0.27833
Erythronic acid	0.20818	-0.29601	-0.68071
Threonic acid	0.868048	-0.22018	-0.30234
Total lactones of threonic and erythronic acids	0.846503	-0.05398	-0.09043
Gluconic acid	0.765791	-0.09955	-0.16276
6-phosphogluconic acid	0.610632	-0.17823	-0.20584
2-ketogluconic acid	0.19898	-0.14009	-0.17217
Valine	0.808511	-0.34706	-0.34609
Leucine + Isoleucine	0.771645	-0.34467	-0.24469
Threonine	-0.11442	-0.57716	0.093483
Methionine	-0.52704	-0.15887	0.363091
Phenylalanine	-0.71508	0.333066	-0.24174
Tryptophan	-0.47757	-0.59347	-0.16255
Lysine + Histidine	-0.44568	0.423899	0.331434
Arginine + Citrulline	-0.72705	-0.10099	0.048552
α-Alanine	0.6877	0.179179	0.326194
Glycine	-0.33508	0.219084	-0.30073
Proline	0.765797	-0.05777	-0.00876
Serine	0.879865	-0.23086	0.034734
Tyrosine	-0.54798	-0.3992	-0.19066
Aspartic acid	0.704507	-0.16702	0.427203
Asparagine	-0.62343	0.422808	0.202928
Glutamic acid	0.588536	0.081303	0.290364
Glutamine	-0.11602	-0.18513	0.230136
β-Alanine	-0.12034	-0.4435	-0.38829
GABA	-0.56099	-0.10261	-0.29736
Hydroxyproline	0.090713	0.051803	0.358483
Oxoproline	0.60012	-0.28406	-0.20976
β-phenyl-α-alanine	-0.52658	-0.05511	-0.22341
Pipecolic acid	0.450656	-0.30449	0.480204
5-hydroxypipecolic acid	-0.25066	0.164287	-0.31062

Ornithine	-0.73109	0.355966	-0.28796
Ornithine lactam	-0.47887	-0.32055	-0.37375
Ethanolamine	-0.20834	0.630377	0.042034
Glycerol	0.550472	0.117388	0.654013
Threitol + erythritol	-0.54728	-0.09119	0.169697
Arabinitol	-0.37735	0.222879	0.614675
Glycerol-3-phosphate	0.347244	-0.07877	0.461674
Deoxyglucitol	-0.7707	0.168442	0.010178
Sorbitol	-0.86182	-0.06075	-0.10566
Allo-inositol	0.048997	-0.12973	-0.26307
Methyl-inositol	-0.90819	0.01001	-0.14538
Ononitol	-0.73604	-0.43383	-0.02329
Chiro-inositol	-0.61471	-0.08459	-0.22357
Myo-inositol	0.946847	0.002969	0.019274
Galactinols	-0.3671	-0.28393	0.2803
Phytol	-0.73805	0.124392	0.130237
α-tocopherol	-0.47454	-0.31308	0.054826
Fatty acids	-0.32268	0.341682	-0.50897
Acylglycerols	0.344226	-0.34659	0.477657
Total saturated fatty acids	0.136809	0.778197	0.220293
Total unsaturated fatty acids	-0.13681	-0.7782	-0.22029
Campesterol	-0.03836	-0.73428	0.320556
Stigmasterol	-0.8655	0.413786	-0.01726
β-Sitosterol	0.897728	-0.32701	-0.02223
Sterol 486	-0.39891	-0.16996	0.291818
Isofucosterol	0.157893	-0.16823	-0.18627
Pentosa	0.069822	-0.01414	0.311984
Ramnose	-0.53572	-0.34946	-0.00336
Fructose	0.51983	-0.07012	0.411171
Mannose + Galactose	-0.4905	0.164481	0.026144
Glucose + Glucose derivatives	-0.66654	0.062488	-0.10788
Sorbose	-0.66881	-0.19961	0.115218
Sucrose	0.651267	-0.00493	-0.21555
Rutinose + rutinose derivatives	-0.52316	-0.18495	0.455462
Raffinose	-0.28845	-0.11643	0.284726
aC9:0	-0.14272	0.061954	-0.18442
aC11:0	0.207606	0.005461	0.568338
C13:0	0.415797	0.216706	0.522303
C16:0	0.048253	0.703151	0.31633
C18:0	0.443522	0.418728	-0.04292
C20:0	-0.23778	-0.52032	-0.19058
C22:0	-0.26039	0.625426	-0.26238
C24:0	-0.62642	-0.3132	-0.27853
C26:0	-0.59773	-0.18821	0.063597

C28:0	-0.79484	-0.01973	0.099014
C18:1	0.280832	-0.47636	0.042526
C18:2	0.60819	-0.58806	0.048542
C18:3	-0.55925	-0.39024	-0.2874
Expl.Var	29.51966	11.04438	9.131818
Prp.Totl	0.28114	0.10518	0.086970

.

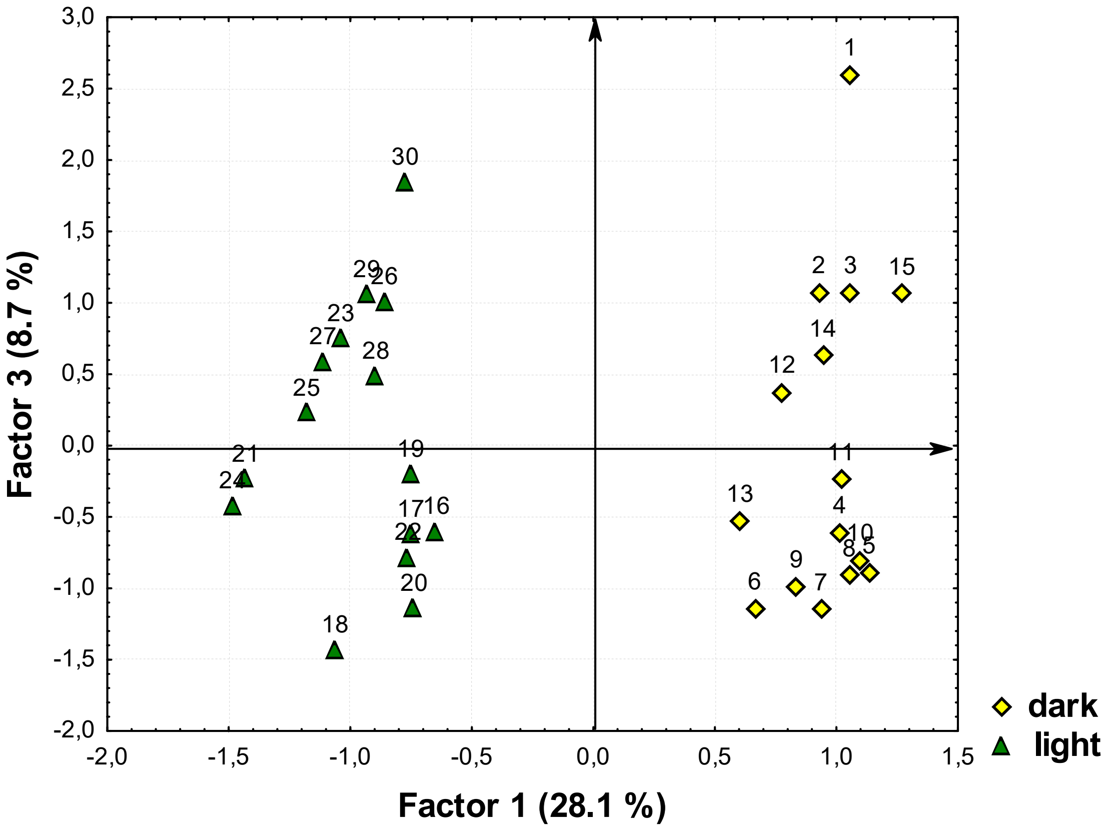

Figure S2. A scatterplot of mung bean sprouts in the space of the first and third factors, calculated according to the proportions of individual substances (%) in the total amount of each identified class of compounds
